# Repeated hybridization of two closely related gazelle species (*Gazella bennettii* and *Gazella subgutturosa*) in central Iran

**DOI:** 10.1002/ece3.6774

**Published:** 2020-09-15

**Authors:** Davoud Fadakar, Mansoureh Malekian, Mahmoud R. Hemami, Hannes Lerp, Hamid R. Rezaei, Eva V. Bärmann

**Affiliations:** ^1^ Department of Natural Resources Isfahan University of Technology Isfahan Iran; ^2^ Natural History Collections Museum Wiesbaden Wiesbaden Germany; ^3^ Department of Fishery and Environment Gorgan University of Agricultural Science and Natural Resources Gorgan Iran; ^4^ Zoological Research Museum Alexander Koenig Bonn Germany

**Keywords:** chinkara, conservation genetics, cytochrome *b*, desert ungulate, goitered gazelle, hybridization, mitochondrial capture, nuclear intron

## Abstract

Interspecific hybridization increasingly occurs in the course of anthropogenic actions, such as species translocations and introductions, and habitat modifications or occurs in sympatric species due to the shortage of conspecific mates. Compared with anthropogenically caused hybridization, natural hybridization is more difficult to prove, but both play an important role in conservation. In this study, we detected hybridization of two gazelle sister species, *Gazella bennettii* (adapted to dry areas) and *Gazella subgutturosa* (adapted to open plains), in five habitat areas, where *G. bennettii* naturally occur in central Iran. The hybrids have a nuclear genomic identity (based on two introns), habitat preference, and phenotype of *G. bennettii*, but the mitochondrial identity (based on cyt *b*) of *G. subgutturosa*. We suggest that natural hybridization of female *G. subgutturosa* and male *G. bennettii* happened twice in central Iran in prehistoric times, based on the haplotype pattern that we found. However, we found indications of recent hybridization between both species under special circumstances, for example, in breeding centers, due to translocations, or in areas of sympatry due to the shortage of conspecific mates. Therefore, these two species must be kept separately in the breeding centers, and introduction of one of them into the habitat of the other must be strictly avoided.

## Graphical abstract

The hybrids have the nuclear identity (based on two introns), habitat, and phenotype of *Gazella bennettii*, and the mitochondrial identity (based on cyt *b*) of *Gazella subgutturosa*. These two species must be kept separately in the breeding centers, and introduction of one of them into the habitat of the other must strictly be avoided.

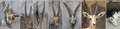

## 1 INTRODUCTION

Translocations and introductions of organisms with an unknown genetic makeup as well as habitat modifications by humans are the major causes of sharply increasing rates of hybridization and introgression, which might ultimately lead to the extinction of species (Allendorf, Leary, Spruell, & Wenburg, 2001; Rhymer & Simberloff, 1996; Vonlanthen et al., 2012). Due to the human‐mediated global change, also natural hybridization of closely related species might increase and play an increasing role in species evolution (Allendorf et al., 2001; Cabria et al., 2011; Dowling & Secor, 1997; Gardner, 1997; Grant & Grant, 1992; Kingston & Gwilliam, 2007; Rhymer & Simberloff, 1996).

Interbreeding between wild species and their domestic forms (Godinho et al., 2011; Hedrick, 2009; Oliveira, Godinho, Randi, & Alves, 2008) and artificially managed or introduced species outside their natural range (Green & Rothstein, 1998; Grobler et al., 2011; van Wyk, Kotzé, Randi, & Dalton, 2013) are the most frequent anthropogenic causes of hybridization in mammals. Furthermore, following Hubbs’ principle or “desperation hypothesis” (Hubbs, 1955), natural hybridization between rare and common sympatric species can occur due to the shortage of conspecific mates (Cabria et al., 2011; Cordingley et al., 2009; Lancaster, Gemmell, Negro, Goldsworthy, & Sunnucks, 2006; Vaz Pinto, Beja, Ferrand, & Godinho, 2016; Willis, Crespi, Dill, Baird, & Hanson, 2004).

In ungulate species, several studies described anthropogenically caused hybridization between native red deer (*Cervus elaphus*) and introduced Japanese sika deer (*Cervus nippon*) in the UK (Abernethy, 1994; Goodman, Barton, Swanson, Abernethy, & Pemberton, 1999; McDevitt et al., 2009; Pérez‐Espona, Pemberton, & Putman, 2009; Senn & Pemberton, 2009), and hybridization was also reported between black wildebeest (*Connochaetes gnou*) and blue wildebeest (*Connochaetes taurinus*) in forced sympatry (Ackermann, Brink, Vrahimis, & De Klerk, 2010; Grobler et al., 2005, 2011), as well as between giant sable antelope (*Hippotragus niger variani*) and roan antelope (*Hippotragus equinus*) in southern Africa in an area where both species were extremely rare (Vaz Pinto et al., 2016). In the genus *Gazella*, the only record of hybridization until now is the introgression of mitochondrial DNA between *G. marica* and *G. subgutturosa* in eastern Turkey (Murtskhvaladze, Gurielidze, Kopaliani, & Tarkhnishvili, 2012). Morphologically, intermediate individuals, probably natural hybrids between the two nominal forms (Groves, 1997; Groves & Harrison, 1967; Mallon & Kingswood, 2001), occur in a vast area between the Tigris/Euphrates valley and the Zagros Mountains, but the only molecular study that included samples from this region (five captive individuals from the Rutba region in Iraq — Wacher et al., 2010) only detected mitochondrial sequences of *G. subgutturosa*. Detection of natural or anthropogenic hybridization is often difficult, but important for conservation actions because of the complex situation of policies and management decision for hybrids (Allendorf et al., 2001; Ellstrand et al., 2010; Stronen & Paquet, 2013; Trouwborst, 2014).

Iran is home to three, possibly four gazelle species and can be regarded as a hotspot for gazelle diversity. Arabian mountain gazelles (*Gazella arabica dareshurii*) are restricted to Farur Island in the Persian Gulf. In southwestern Iran, populations of sand gazelles (*G. marica*) exist based on mitochondrial cytochrome *b* (cyt *b*) sequences (Fadakar et al., 2019), but morphologically these individuals are intermediate in size and morphology between *G. marica* and *G. subgutturosa* and might be natural hybrids (Groves & Harrison, 1967), which remains to be confirmed using nuclear DNA. Goitered gazelles (*G. subgutturosa*) and chinkara (*G. bennettii*) occur in large parts of the country. Their ranges meet in central Iran, where both species occur in neighboring areas (e.g., *G. subgutturosa* in Kalmand‐Bahadoran Protected Area and *G. bennettii* in Darre Anjir Wildlife Refuge).

*Gazella subgutturosa* is associated with open plains and is widely distributed in all steppes or semi‐deserts, occurring in a relatively high number of individuals (Firouz, 2005; Karami, Hemami, & Groves, 2002). Its sister species, *G. bennettii*, adapted to dry deserts with the ability to survive with very little brackish water in hot weather in the deserts of central Iran, such as Dasht‐e Lut and Dasht‐e Kavir deserts, with a relatively low number of individuals (often less than 50) in each area (Akbari, Moradi, Sarhangzadeh, & Esfandabad, 2014). The two species can easily be differentiated, as females of *G. subgutturosa* are usually hornless (some females may have short and deformed horns), while *G. bennettii* females have long, slender horns. In males, the horns of *G. subgutturosa* are much more out bowed, while males of *G. bennettii* have relatively straight horns. In both sexes, the body size of *G. bennettii* is much smaller than *G. subgutturosa*.

Due to the highly sculptured landscape, intraspecific patterning was detected in *G. subgutturosa* (Khosravi et al., 2018), where two subspecies were found to occur in Iran (Fadakar et al., 2020). *G. bennettii* was also supposed to split into at least two subspecies or even species in Iran (Groves & Grubb, 2011), but this has not been confirmed using molecular data yet. For a study on the genetic diversity of *G. bennettii*, we therefore collected fecal and tissue samples of this species throughout the country (Fadakar, unpublished data). In a first step, these samples were sequenced for mitochondrial DNA (cyt *b*) to confirm species identification. Surprisingly, however, some of them were identified as belonging to *G. subgutturosa*, despite being collected in habitats of *G. bennettii* and/or from individuals that were morphologically identified as belonging to *G. bennettii* (see Methods). Therefore, we hypothesize that these samples with *G. subgutturosa* mitochondrial identity belong to hybrids of *G. subgutturosa* and *G. bennettii*. Using additional sequences for two nuclear introns, we aimed at investigating this hypothesis. Furthermore, we asked whether hybridization occurred repeatedly or only once, and whether this is an ongoing process or a prehistoric event.

## 2 MATERIAL AND METHODS

### 2.1 Sampling

**FIGURE 1:**
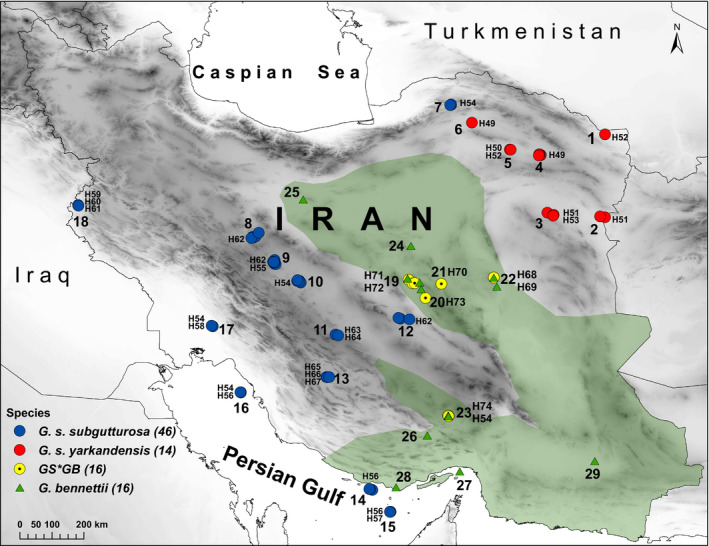
Sample locations of *Gazella subgutturosa* subspecies, *Gazella bennettii*, and hybrid samples in Iran. 1 = Jangal Khajeh PA (KHJE), 2 = Khaf Breeding Center (KHAF), 3 = Hengam PA (HNGM), 4 = Reisi PA (REIS), 5 = Shir Ahmad WR (SHIR), 6 = Miandasht WR (MNDT), 7 = Golestan NP (GLSN), 8 = Mooteh WR (MOTE), 9 = Qamishlou NP (QMIS), 10 = Kolah Ghazi NP (KOLA), 11 = Abadeh PA (ABAD), 12 = Kalmand‐Bahadoran PA (KALM), 13 = Bamu NP (BAMU), 14 = Kish Island (KISH), 15 = Siri Island (SIRI), 16 = Kharg Island (KHRG), 17 = Dimeh PA (DIME), 18 = Qaraviz HPA (QRVZ), 19 = Darre Anjir WR (DNJR), 20 = Ariz HPA (ARIZ), 21 = Bahabad HPA (BHBD), 22 = Naybandan WR (NBND), 23 = Khabr NP (KHBR), 24 = Abas Abad WR (ABAB), 25 = Kavir NP (KVIR), 26 = Tarom PA (TARM), 27 = Hormoz Island (HRMZ), 28 = Bandar‐e Lengeh (BLNG), 29 = Bazman HPA (BAZM). The distribution area of *G. bennettii* in Iran is indicated by a green polygon

In total, 32 samples, collected in *G. bennettii* habitats and stored in 96% ethanol, are included in this study (Table 1 and Figure 1), including 21 fecal and 11 tissue samples.

**TABLE 1:** List of gazelle samples used in this study, with cyt *b* haplotype (Hap – see Table S1), GenBank accession numbers for cyt *b* and nuclear intron (ZNF618 and CHD2) sequences, sample location, and type of sample

No	Species	ID	Hap	Cyt *b*	ZNF618	CHD2	Location	S_type
1	*G. bennettii*	NBND1	HB2	MT811627	MT822208	MT822237	South Khorasan, Naybandan WR	Feces
2	*G. bennettii*	NBND8	HB3	MT811628	MT822209	MT822238	South Khorasan, Naybandan WR	Tissue
3	*GS* × *GB*	DoE5	H69	MT811610	—	—	South Khorasan, Naybandan WR	Tissue
4	*GS* × *GB*	NBND10	H69	MT811611	MT822210	MT822239	South Khorasan, Naybandan WR, BC	Tissue
5	*GS* × *GB*	NBND2	H68	MT811607	MT822211	MT822240	South Khorasan, Naybandan WR, BC	Tissue
6	*GS* × *GB*	NBND3	H68	MT811608	—	—	South Khorasan, Naybandan WR, BC	Tissue
7	*GS* × *GB*	NBND4	H68	MT811609	MT822212	MT822241	South Khorasan, Naybandan WR, BC	Tissue
8	*GS* × *GB*	NBND5	H69	MT811612	MT822213	MT822242	South Khorasan, Naybandan WR, BC	Tissue
9	*GS* × *GB*	NBND7	H69	MT811613	MT822214	MT822243	South Khorasan, Naybandan WR, BC	Tissue
10	*G. bennettii*	DNJR1	HB1	MT811623	MT822215	MT822244	Yazd, Darre Anjir WR	Feces
11	*G. bennettii*	DNJR2	HB1	MT811624	MT822216	MT822245	Yazd, Darre Anjir WR	Feces
12	*G. bennettii*	DNJR6	HB1	MT811625	MT822217	MT822246	Yazd, Darre Anjir WR	Feces
13	*GS* × *GB*	DNJR3	H71	MT811616	MT822218	MT822247	Yazd, Darre Anjir WR	Feces
14	*GS* × *GB*	DNJR4	H72	MT811618	MT822219	MT822248	Yazd, Darre Anjir WR	Feces
15	*GS* × *GB*	DNJR5	H71	MT811617	—	—	Yazd, Darre Anjir WR	Tissue
16	*G. bennettii*	ARIZ1	HB1	MT811626	MT822220	MT822249	Yazd, Ariz HPA	Feces
17	*GS* × *GB*	ARIZ2	H73	MT811619	—	MT822250	Yazd, Ariz HPA	Feces
18	*GS* × *GB*	BHBD1	H70	MT811614	—	—	Yazd, Bahabad HPA	Tissue
19	*GS* × *GB*	BHBD2	H70	MT811615	—	—	Yazd, Bahabad HPA	Tissue
20	*G. bennettii*	KHBR1	HB4	MT811629	MT822221	MT822251	Kerman, Khabr NP	Feces
21	*G. bennettii*	KHBR14	HB5	MT811631	MT822222	MT822252	Kerman, Khabr NP	Feces
22	*G. bennettii*	KHBR15	HB5	MT811632	MT822223	MT822253	Kerman, Khabr NP	Feces
23	*G. bennettii*	KHBR2	HB6	MT811630	—	MT822254	Kerman, Khabr NP	Feces
24	*GS* × *GB*	KHBR12	H74	MT811620	—	MT822255	Kerman, Khabr NP	Feces
25	*GS* × *GB*	KHBR16	H74	MT811621	—	—	Kerman, Khabr NP	Feces
26	*GS* × *GB*	KHBR3	H54	MT811622	—	MT822256	Kerman, Khabr NP	Feces
27	*G. bennettii*	TARM	HB6	MT811638	MT822224	MT822257	Fars, Tarom PA	Feces
28	*G. bennettii*	BLNG2	HB8	MT811635	MT822225	MT822258	Hormozgan, Bandar‐e Lengeh	Feces
29	*G. bennettii*	HRMZ1	HB9	MT811637	MT822226	MT822259	Hormozgan, Hormoz Island	Feces
30	*G. bennettii*	BIAZ1	HB1	MT811633	MT822227	MT822260	Isfahan, Abas Abad WR	Feces
31	*G. bennettii*	KVIR3	HB7	MT811636	MT822228	MT822261	Semnan, Garmsar, Kavir NP	Feces
32	*G. bennettii*	IRSH1	HB1	MT811634	MT822229	MT822262	Sistan Balouchestan, Bazman HPA	Tissue
33	*G. subgutturosa*	QAMS3	H55	MT264061	MT822230	MT822263	Isfahan, Qamishlou NP	Feces
34	*G. subgutturosa*	MOTE6	H62	MT264070	MT822231	MT822264	Isfahan, Mooteh WR	Feces
35	*G. subgutturosa*	QRVZ3	H60	MT264069	MT822232	MT822265	Kermanshah, Qaraviz HPA	Feces
36	*G. subgutturosa*	KHAF1	H51	MT264046	MT822233	MT822266	Razavi Khorasan, Khaf BC	Feces
37	*G. subgutturosa*	KHAF2	H51	MT264047	MT822234	MT822267	Razavi Khorasan, Khaf BC	Feces
38	*G. subgutturosa*	SHIR1	H50	MT264043	MT822235	MT822268	Razavi Khorasan, Shir Aahmad WR	Feces
39	*G. subgutturosa*	KALM6	H62	MT264083	MT822236	MT822269	Yazd, Kalmand‐Bahadoran PA	Feces

Abbreviations: BC, breeding center; DoE, Department of Environment; *GS* × *GB*, *G. subgutturosa* × *G. bennettii* hybrids; HPA, Hunting Prohibited Area; NP, National Park; PA, Protected Area; WR, Wildlife Refuge.

**FIGURE 2:**
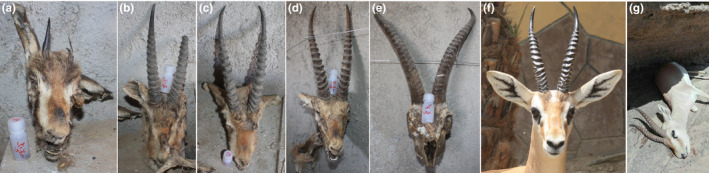
Hybrid samples in the breeding center of Naybandan WR (a–d), typical *Gazella subgutturosa* of the breeding center of Naybandan WR (e), typical pure *Gazella bennettii* in Bahou Kalat WR at southeast Iran (f), and hybrid sample from Darre Anjir WR which died due to snakebite (g). (e and f) are presented here to show phenotypical trait differentiation between typical pure *G. bennettii* and *G. subgutturosa*

Six tissue samples were collected from dried heads (Figure 2a–d) of individuals that were killed by a caracal (*Caracal caracal*) in the breeding center of Naybandan Wildlife Refuge (WR; A. Mirkalani, personal communication), and another one from this region stored at the museum of the Department of Environment. Two tissue samples were provided by the Yazd Department of Environment from confiscated illegal hunting in Bahabad Hunting Prohibited Area (HPA) in Bahabad desert. The desert is located between Bahabad city in the south, Naybandan WR in the east, Robat‐e Posht‐e Badam in the north, and Ardakan city in the west. One tissue sample was collected from a dead animal in Darre Anjri WR (Figure 2g), and one from a carcass found in Bazman HPA (southeastern Iran). All fresh feces were collected in the field, after observing putative *G. bennettii* from a distance allowing morphological species identification.

Additional fecal samples of *G. subgutturosa* came from Razavi Khorasan Province (northeastern Iran), Isfahan and Yazd Provinces (central Iran), and Kermanshah Province (western Iran). These were previously sequenced for cyt *b* by Fadakar et al. (2020). The locality information, species identity, and the kind of material of all samples are summarized in Table 1.

### 2.2 DNA extraction, amplification, and sequencing

Whole genomic DNA was extracted from samples using AccuPrep genomic DNA extraction kit (Bioneer) following the manufacturer's instructions. Polymerase chain reaction (PCR) was performed for amplification of the complete coding region of the cyt *b* gene of mtDNA using CYTB_F (5′‐CCCCACAAAACCTATCACAAA‐3′) and CYTB_R (5′‐AGGGAGGTTGGTTGTTCTCC‐3′) primers (Pedrosa et al., 2005; Rezaei et al., 2010). The reaction mixture was prepared in 25 μl volume, containing 1 unit of Euro Taq DNA polymerase, 10 µM Tris‐HCl, 30 µM KCl, 1.5 mM MgCl_2_, 250 µM of each dNTP, and 2 pmol primers (Bioneer).

The thermocycling for CYTB_F and CYTB_R primers we used the following protocol (Rezaei et al., 2010): 10 min at 95°C followed by 35 cycles of 30 s at 95°C, 30 s at 55°C, and 60 s at 72°C, and finally followed by 7 min at 72°C. Sanger sequencing was performed using the BigDye Terminator Cycle Sequencing kit v.3.1 (Applied BioSystems) and electrophoresis of the purified sequencing product was carried out on an ABI PRISM 3730xl automatic sequencer. Sequences were edited for correction with SeqScape v.2.6 software (Applied Biosystems). All new sequences were preliminarily identified using a BLAST search against known gazelle sequences on GenBank and were submitted to GenBank (MT811607‐MT811638, Table 1).

### 2.3 Nuclear markers

For a phylogenetic analysis of the genus *Gazella*, Lerp et al. (2016) published a new set of nuclear intron markers. These had only limited variation, but showed very high consistency in the delineation of species. Only two of the six markers (chromodomain–helicase–DNA‐binding protein 2 (CHD2) and zinc finger protein 618 (ZNF618)) were able to differentiate between *G. subgutturosa* and *G. bennettii*, and both were sequenced for all 32 samples of putative *G. bennettii* and seven pure *G. subgutturosa* using the primers from Lerp et al. (2016). The PCR was carried out in a GeneAmp 2720 Thermo Cycler (Applied Biosystems) using QIAGEN Multiplex PCR Kit in 20 μl volume, containing 2 µl Q‐Solution, 10 µl QIAGEN Multiplex PCR Master Mix (including HotStarTaq DNA Polymerase, QIAGEN Multiplex PCR Buffer, and dNTP Mix), and 1.6 µl of each primer (10 pmol/µl) using the following protocol: 15 min at 95°C (initial step), followed by 38 cycles of 35 s at 95°C, 60 s at 60°C, and 60 s at 72°C, and finally 10 min at 72°C (final elongation). PCR products were purified using 6 µl of HT ExoSAP‐IT (Thermo Scientific). Purified PCR products were send off to Macrogen for Sanger Sequencing. The new sequences were aligned with previously published sequences of the genus *Gazella* (Lerp et al., 2016) using the Clustal W algorithm (Thompson, Higgins, & Gibson, 1994) implemented in Mega v.5 (Tamura et al., 2011). New sequences were submitted to GenBank (MT822208–MT822269, Table 1).

### 2.4 Haplotype network for cyt *b*

A median‐joining (MJ) network was constructed for 295 sequences, including 16 new complete cyt *b* sequences from supposed hybrid individuals (this study), 60 sequences of *G. subgutturosa* from Iran (H49–H67 – Fadakar et al., 2020), and 219 published *G. subgutturosa* sequences from Asia (H1–H48 – Abduriyim, Nabi, & Halik, 2018; Dong et al., 2016; Hassanin et al., 2012; Hassanin & Douzery, 1999; Lerp et al., 2016) using the software PopART v.1.7 (Leigh & Bryant, 2015) with the default settings (Table S1, see Fadakar et al., 2020 for details of the alignment).

### 2.5 Phylogenetic analysis

For the phylogenetic analysis of mitochondrial cyt *b* sequences, we used sequences of all haplotypes collected from the habitats of *G. bennettii*, as well as one previously published sequence of *G. bennettii* (NC020703) and all haplotype sequences of *G. subgutturosa* from GenBank. Four sequences from closely related sand gazelle (*G. marica*) and slender‐horned gazelle (*Gazella leptoceros*) were used as outgroup representatives (Table S1). Sequences were aligned using the Clustal W algorithm (Thompson et al., 1994) implemented in Mega v.5 (Tamura et al., 2011), and final adjustments were made by eye.

The best‐fitting partitioning scheme and nucleotide substitution models were estimated using greedy search algorithm with PhyML (Guindon et al., 2010) in PartitionFinder v.2.1.1 (Lanfear, Calcott, Ho, & Guindon, 2012; Lanfear, Frandsen, Wright, Senfeld, & Calcott, 2017). We tested among partitioning schemes including division of protein‐coding genes into 1st, 2nd, and 3rd codon positions. Models were selected by the Bayesian information criterion (BIC). We found the optimal partitioning scheme includes three partitions (optimal models are indicated in brackets) 1st codon (K80 + G), 2nd codon (HKY + I), and 3rd codon (GTR + I). Bayesian interference analyses were carried out in MrBayes v.3.2 (Ronquist et al., 2012) with two independent runs of four Markov chains (one cold and three heated) over 10,000,000 generations and sampling every 1,000 generations. The first 25% of the sampled trees and estimated parameters were discarded as burn‐in. Convergence of the model parameters was monitored using the program Tracer v.1.7.1 (Rambaut, Drummond, Xie, Baele, & Suchard, 2018). The consensus phylogenetic tree was then edited in FigTree v.1.4.4 (http://tree.bio.ed.ac.uk/software/figtree/).

## 3 RESULTS

### 3.1 Sequencing and mitochondrial cyt *b*

Extracting and amplifying mitochondrial and even nuclear DNA sequences from fecal samples was unproblematic, but from some tissue samples from Naybandan WR we obtained no results, presumably because of improper storing conditions (in a shed) for a long time. Using cyt *b*, 16 of 32 samples of putative pure *G. bennettii* were identified as *G. subgutturosa* in a BLAST search against known gazelle sequences on GenBank. These are therefore hypothesized to belong to hybrids of *G. bennettii* and *G. subgutturosa* in the subsequent analyses.

### 3.2 Nuclear intron markers

Sequencing of CHD2 (652 bp) and ZNF618 (676 bp) for putative hybrid specimens was successful for samples from Darre Anjir WR (two), Ariz HPA (one, only CHD2), Khabr NP (two, only CHD2), and Naybandan WR (five), see Table 1. Also, sequencing for all pure *G. bennettii* (except ZNF618 for KHBR2) and all *G. subgutturosa* individuals was successful.

For CHD2 the only difference between *G. bennettii* and *G. subgutturosa* is a mutation at position 221 (“C” in *G. subgutturosa*, *G. marica*, *G. leptoceros*, and *G. cuvieri*; “T” in *G. bennettii* and all other species). This is also the case in our pure *G. subgutturosa* samples, and in all pure *G. bennettii* samples (see Table 1). All sequences from putative hybrids were identified as *G. bennettii* for CHD2.

For ZNF618, three of the five previously published *G. bennettii* samples (from Lerp et al., 2016) show an insertion/duplication of 6 bp after position 212 (from 5′ to 3′) that is not present in any other gazelle species. This insertion is present in most of our pure *G. bennettii* samples: Eight samples are homozygotic for the indel, five are heterozygotic, and two samples (from Bandar‐e Lengeh, and Darre Anjir WR) do not have the indel (one unknown); and in all putative hybrids: five samples are homozygotic for the indel, two are heterozygotic (nine unknown – see Table 2). Heterozygotic indels are inferred from the raw sequencing reads: If the forward and reverse sequences can clearly be read up to the indel sequence but not across, the indel is heterozygotic (the two alleles produced PCR products with different lengths, and therefore, the sequencing results are unreadable from the point onwards where they start to differ).

**TABLE 2:** Variable sites in the intron sequence of ZNF618 (total length 676 bp)

Species	Location	ID	Variable sites					Insertion	Variable sites							
			48	84	108	136	189	213–218	238	175	305	355	398	406	501	607
*GB* × *GS*	Hybrid areas	NBND2	—	G	C	A	C	GTCAGG	C	T	G	G	G	G	C	C
		NBND4	—	.	.	.	.	GTCAGG	.	.	.	.	.	.	.	.
		NBND5	—	.	.	.	.	GTCAGG	.	Y	.	.	.	.	.	.
		NBND7	—	.	.	.	.	GTCAGG	.	.	.	.	.	.	.	.
		NBND10	—	.	.	.	.	GTCAGG*	.	Y	.	.	.	.	.	.
		DNJR3	—	.	.	.	.	GTCAGG	.	.	.	.	.	R	.	.
		DNJR4	—	?	?	?	?	GTCAGG*	T	C	.	.	.	.	.	.
*G. bennettii*	Hybrid areas	DNJR1	—	.	.	.	T	—	T	C	.	.	.	.	.	?
		DNJR2	—	.	.	.	T	GTCAGG*	T	C	.	.	.	.	.	.
		DNJR6	—	.	.	.	.	GTCAGG	.	.	.	.	.	.	.	.
		ARIZ2	—	.	.	.	.	GTCAGG*	.	C	.	.	.	.	.	.
		NBND1	—	.	.	.	.	GTCAGG	.	.	.	.	.	.	.	.
		NBND8	—	.	.	.	.	GTCAGG	.	.	.	.	.	.	.	.
		KHBR1	—	.	.	.	.	GTCAGG	.	C	.	.	.	.	.	.
		KHBR14	—	.	.	.	?	GTCAGG*	T	C	.	.	.	.	.	?
		KHBR15	—	?	?	?	.	GTCAGG*	.	C	.	.	.	.	?	?
	Outside of hybrid areas	BIAZ1	—	.	.	.	.	GTCAGG	.	C	.	.	.	.	.	.
		IRSH1	—	.	.	.	T	GTCAGG*	.	C	.	.	.	.	.	.
		BLNG2	—	.	.	.	.	—	.	C	.	.	.	.	.	.
		KVIR3	—	.	.	.	.	GTCAGG*	.	C	.	.	.	.	.	.
		HRMZ1	—	.	.	.	.	GTCAGG	.	C	.	.	.	.	.	.
		TARM4	—	.	.	.	.	GTCAGG	.	C	.	.	.	.	.	.
	GenBank	KU560859	—	.	.	.	.	GTCAGG	.	.	.	.	.	.	.	.
		KU560857	—	.	.	.	.	GTCAGG	.	C	.	?	?	?	?	?
		KU560856	—	.	.	.	.	GTCAGG	.	C	.	.	R	.	.	.
		KU560858	—	R	.	.	.	—	.	C	.	.	.	.	.	.
		KU560855	—	R	.	.	.	—	.	C	.	.	.	.	.	Y
*G. subgutturosa*	Outside of hybrid areas	SHIR1	—	.	.	.	.	—	.	C	.	.	.	.	.	.
		KHAF1	—	.	.	.	.	—	.	C	R	R	.	.	.	.
		KHAF2	—	.	.	.	.	—	.	C	R	R	.	.	Y	.
		QAMS3	—	.	.	.	.	—	.	C	.	.	.	.	.	.
		QRVZ3	—	.	.	.	.	—	.	C	.	.	.	.	.	.
		KALM6	—	.	T	T	.	—	.	C	A	A	.	.	T	.
		MOTE6	—	.	.	T	.	—	.	C	A	A	.	.	T	.
	GenBank	KU560854	A	.	.	.	.	—	.	C	.	.	.	.	.	.

Note

“Hybrid areas” refer to locations where hybrid specimens (with nucl. identity of *G. bennettii* and mitoch. identity of *G. subgutturosa*) were found to occur. Samples heterozygotic for the indel are marked with “*” in the indel column.

Abbreviation: *GS* × *GB*, *G. subgutturosa* × *G. bennettii* hybrids.

### 3.3 Haplotype network

**FIGURE 3:**
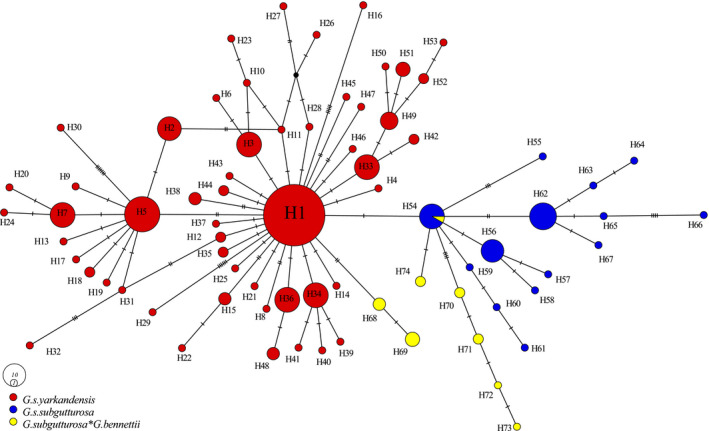
Median‐joining network of cyt *b* sequences of *Gazella subgutturosa* subspecies and *G. subgutturosa* × *Gazella bennettii* hybrids. Mutational steps among haplotypes are signaled with dash lines and small, filled black, circles refer to inferred missing haplotypes. Each circle represents a different haplotype, whereby areas of circles are proportional to the number of sampled individuals (see the legend for the circle sizes of one and ten samples, respectively)

The reconstructed MJ network based on complete cyt *b* provides an overview of the haplotype distribution and relationships within the two *G. subgutturosa* subspecies and the hybrid haplotypes (Figure 3). Samples from Bahabad HPA, Darre Anjir WR, Ariz HPA (all three are located in Yazd Province), and Khabr NP (Kerman Province) are connected with (or even identical to) H54, the supposed ancestral haplotype of *G. subgutturosa* that belongs to the nominate subspecies *G. subgutturosa subgutturosa* (Fadakar et al., 2020). Samples from Naybandan WR (South Khorasan Province) derived from H1, the most frequent haplotype of the *G. s. yarkandensis* subspecies (Figure 3). Of the eight haplotypes found in hybrids, only one has been detected in pure *G. subgutturosa* specimens: the ancestral haplotype H54 is present in hybrids in Khabr NP. All other hybrid haplotypes are new (H68–H74).

### 3.4 Phylogeny

**FIGURE 4:**
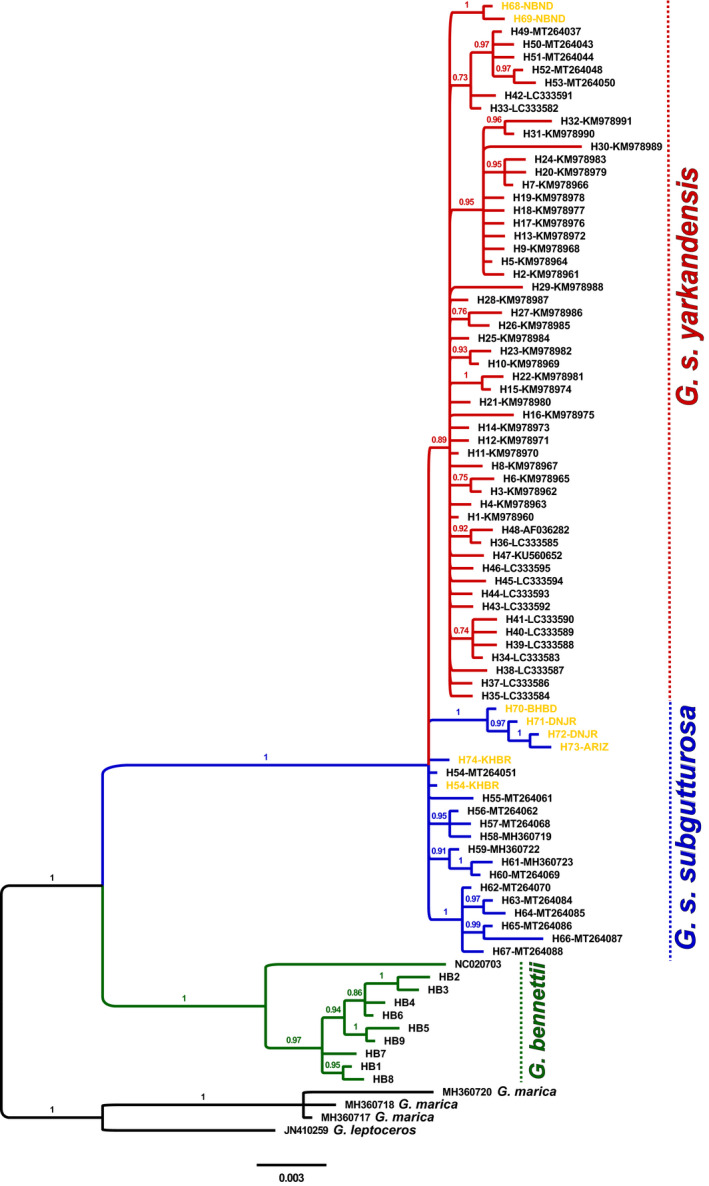
Phylogeny of *Gazella subgutturosa*, *Gazella bennettii* (green), and *G. subgutturosa* × *G. bennettii* hybrids (yellow) from Bayesian analysis of complete cyt *b* gene sequences. The trees were summarized with the majority‐rule consensus tree. Numbers above branches are posterior probabilities

The phylogenetic analysis of complete cyt *b* sequences resolves *G. subgutturosa* and *G. bennettii* as sister species (Figure 4) as expected from previous studies (Abduriyim et al., 2018; Bärmann, Rössner, & Wörheide, 2013; Dong et al., 2016; Fadakar et al., 2019, 2020; Fadakar et al., 2013; Khosravi, Malekian, Hemami, Silva, & Brito, 2019; Lerp et al., 2016; Wacher et al., 2010). Samples from the central Iranian locations are either placed as *G. bennettii* or as *G. subgutturosa* (supposed hybrids). Similar to the haplotype network, the hybrid samples fall into two distinct clades, one within *G. s. yarkandensis* (PP = 0.91) and one within *G. s. subgutturosa*, but the latter are not resolved as forming a monophyletic group as one sample has the ancestral *G. subgutturosa* haplotype H54.

## 4 DISCUSSION

Our results clearly show that the deserts in central Iran, including Darre Anjir WR, Bahabad HPA, Ariz HPA, Naybandan WR, and Khabr NP, are home to a group of gazelles with mixed ancestry, that is, nuclear identity of *G. bennettii* (based on two introns) and mitochondrial identity of *G. subgutturosa* (based on cyt *b*). Until now, only *G. bennettii* was commonly recorded in these areas, although there have been very rare direct observations of *G. subgutturosa* from close distance, for example, one adult female with a calf in Naybandan WR in 2010 (A. Mirkalani, personal communication), one adult female with a calf in Ariz HPA in winter 2017, and several observations of *G. subgutturosa* in “Kalout‐e Abi Area” near Bafq Protected Area (PA), which is close to Darre Anjir WR and Ariz HPA between 2012 and 2017 (A. Khajavi, personal communication). In Khabr NP, there were never any observations of *G. subgutturosa*. This area is considered the best habitat of *G. bennettii* in Iran with more than 1,000 resident individuals.

Introgression of mitochondrial DNA among closely related gazelles has been demonstrated for *G. marica* and *G. subgutturosa* (Murtskhvaladze et al., 2012). It could be a more wide‐spread phenomenon in other species of this relatively young genus (2.5 Mya — Bibi, 2013). Our results show that there has been introgression of mitochondrial DNA from *G. subgutturosa* to *G. bennettii* in populations in central Iran, that is, there has been hybridization of female *G. subgutturosa* with male *G. bennettii*. Phenotypically (horn and face pattern, body size) and ecologically (in terms of habitat preference), the hybrid individuals resemble *G. bennettii*, and also the two analyzed nuclear intron markers show *G. bennettii* identity. The hybrid individuals were found in sympatry with pure *G. bennettii* in all areas; therefore, it seems that they belong to mixed populations of pure and hybrid individuals.

The distribution of hybrid haplotypes in the haplotype network of *G. subgutturosa* (Figure 3) shows that hybrid haplotypes from South Khorasan Province (H68 and H69) belong to the *G. subgutturosa yarkandensis* subspecies in northeastern Iran, while those from Yazd Province and Kerman Province (H70–H74, and one individual with the ancestral *G. subgutturosa* haplotype H54) belong to the nominate subspecies in central and southwestern Iran. It is very likely therefore that hybridization occurred twice, each time involving a female of a different subspecies of *G. subgutturosa*.

### 4.1 Hybridization and chromosome numbers

Hybridization in gazelles is probably restricted to very closely related species, as Robertsonian translocations are very frequent in the genome of gazelles (Vassart, Granjon, & Greth, 1995). These are major chromosomal changes where two acrocentric chromosomes fuse to form one biarmed chromosome. If individuals with two different chromosome configurations interbreed, the offspring may have reduced fertility as the chromosomes cannot properly be segregated during meiosis, leading to unbalanced gametes (Baker & Bickham, 1986; Benirschke & Kumamoto, 1991). The ancestral condition in bovids probably is an autosome number of 2*n* = 58 (not including sex chromosomes — Wurster & Benirschke, 1968). While most of the ancestral autosomes are still identifiable in gazelles using banding techniques, many of them have fused to form biarmed chromosomes (including a common X‐to‐autosome‐5 fusion), so the chromosome numbers in the genus *Gazella* are as low as 2*n* = 30–35 (Groves & Grubb, 2011; Vassart et al., 1995 and references therein). The chromosome numbers of *G. subgutturosa*, 2*n* = 31 in males, 2*n* = 30 in females (Schreiber & Hegel, 1999), are in the range of other gazelle species. In contrast, *G. bennettii* has much larger chromosome numbers, that is, 2*n* = 49–51 in males and 2*n* = 50–52 in females (Furley, Tichy, & Uerpmann, 1988; Kumamoto, Kingswood, Rebholz, & Houck, 1995). It is surprising therefore to find hybrids of the two species that seem to be fertile and interbreeding with pure *G. bennettii* in the wild. Interspecific hybrids in other bovid species (e.g., Alcelaphines, Tragelaphines) with (and without) different chromosome numbers were found to be infertile, at least in males (Robinson, Cernohorska, Schulze, & Duran‐Puig, 2015; Vaz Pinto et al., 2016).

### 4.2 Hybridization as a prehistoric event

Hybridization can be a prehistoric phenomenon that might lead to mitochondrial capture of a whole species, for example, as demonstrated for polar bears (*Ursus maritimus* – Hailer et al., 2012) or European bison (*Bison bonasus*) which is very closely related to American bison (*Bison bison*) based on nuclear DNA, but seems to have adapted the mitochondrial identity of some extinct bovine species from Europe (Hassanin, An, Ropiquet, Nguyen, & Couloux, 2013). Also, natural hybridization can occur repeatedly, for example, among *Ovis orientalis* and *Ovis vignei* in the Central Alborz Mountain Range in Iran where hybrids were shown to have intermediate chromosome numbers and phenotypical traits (e.g., horn morphology and coat pattern) of both species (Rezaei et al., 2010).

Although *G. subgutturosa* females have occasionally been observed in the areas with hybrid individuals (see above), the results indicate that the hybridization of *G. subgutturosa* and *G. bennettii* is not a recent event or ongoing process. The cyt *b* haplotypes of pure *G. subgutturosa* and hybrid individuals are (almost) mutually exclusive: Apart from the ancestral H54, all other haplotypes of the hybrid specimens are unique and were not recorded in any population of *G. subgutturosa* so far. Furthermore, the haplotypes of *G. subgutturosa* that are geographically closest to the areas with hybrid individuals are H62 in central Iran (all areas except Khabr NP), as well as H51 and H53 in northeastern Iran (only for Naybandan WR). None of these *G. subgutturosa* haplotypes were found to occur in hybrid individuals, which would be expected if repeated hybridization took place with neighboring pure populations of *G. subgutturosa*.

*Gazella subgutturosa* experienced a strong bottleneck in Iran during the last decades (Hemami & Groves, 2001; Khosravi et al., 2018), so it is possible that the haplotypes involved in hybridization have been extirpated from the pure *G. subgutturosa* populations. Furthermore, small populations of *G. subgutturosa* still exist in unprotected and protected areas not sampled so far. Some of these are relatively close to the areas where hybrids were confirmed, for example, Biduiyeh PA (Kerman Province, approximate population size = 150) from where individuals might be able to migrate to Khabr NP (Khosravi et al., 2018). It could therefore be possible that hybrid haplotypes are present in small *G. subgutturosa* populations not sampled so far. This should be tested by sampling more individuals from hitherto unsampled small populations close to the hybrid populations.

With the current data, however, we conclude that the populations evolved independently after the initial hybridization events and that there was no back‐crossing with *G. subgutturosa* (at least not in the maternal line). Especially in northeastern Iran, it is interesting to see that the hybrid specimens have haplotypes (H68 and H69) that are only two to three mutational steps away from the proposed ancestral haplotype of the *G. s. yarkandensis* subspecies (H1, not present in Iran but reported from China (Fadakar et al., 2020)), but four to five steps away from the pure *G. s. yarkandensis* individuals (H49–H53) also living in Khorasan Province (Figure 3). So the natural hybridization occurred relatively early, probably when the subspecies were not even properly distinct.

With regard to *G. bennettii*, it might even be concluded that the hybridization took place before the chromosome numbers started to change drastically in this species. *G. subgutturosa* and *G. bennettii* are sister species, so it can be assumed that their chromosome numbers were initially identical, which would facilitate hybridization. If hybridization occurred early the hybrids are expected to have the same number of chromosomes as pure *G. bennettii*. If it occurred later, there would be intermediate chromosome numbers in hybrid specimens, which might affect their reproductive success. In any case, it would be extremely interesting to get information on chromosome numbers of the mixed populations to learn more about the evolution of Robertsonian translocations and their influence on speciation processes.

### 4.3 Recent natural hybridization

Hybridization of *G. bennettii* and *G. subgutturosa* might still be possible in the wild, in areas where both species are sympatric with low individual numbers. In Bahabad HPA, for example, the total number of gazelle individuals (*G. bennettii* and *G. subgutturosa*) probably is below 10. Under these conditions, mating between the two species could take place as conspecific mates are not present or hard to find, based on the “desperation hypothesis” (Hubbs, 1955). This could potentially lead to strong outbreeding depression (Randi, 2008), as hybrids might have reduced fertility. The hybrids in Khabr NP could potentially be explained by the “desperation hypothesis”: Khabr NP is located far away from the other *G. subgutturosa* populations sampled by Fadakar et al. (2020), but previously there has been a small population of *G. subgutturosa* in the northern part of Khabr NP. In order to increase the population of *G. bennettii*, the core area of the National Park was fenced about three decades ago. After fencing, the population of *G. bennettii* increased sharply (from about 70 to over 1,000), while the *G. subgutturosa* population decreased dramatically because their habitat was outside the fenced area of the National Park and severely under the pressure of illegal hunting, competition with livestock and habitat destruction which eventually led to their extinction. However, within the fenced area, remnant individuals of *G. subgutturosa* might have mixed with the abundant population of *G. bennettii*. This would have been a rather recent hybridization, which could explain why there is still a recent haplotype of *G. subgutturosa* (H54) present in the hybrids. However, as the *G. bennettii* population has been strongly increasing, there seems to be no negative effect of this hybridization on the reproduction and viability of the mixed population, which would be expected according to the very different haplotype numbers of the two parent species. Other reported cases of interspecific hybridization in bovids have shown that even if the parent species have different chromosome numbers, female offspring can be fertile, for example, in an *Alcelaphus buselaphus* × *Damaliscus lunatus* cross reported by Robinson et al. (2015). So maybe one fertile female hybrid was enough to keep the *subgutturosa* haplotype in the *G. bennettii* population. In any case, an investigation in chromosome numbers would be desirable especially for individuals in this mixed population.

If, however, recent hybridization occurs repeatedly, we expect to see a negative effect on the population viability of the (hybrid‐)*G. bennettii* population. One potential case of this is Darre Anjir WR, and other protected areas in central Iran. Although the level of protection has increased, there, the population numbers of *G. bennettii* (and “old” hybrids), have not increased (Akbari et al., 2014). One possible explanation might be that *G. subgutturosa* individuals occasionally migrate from Kalmand‐Bahadoran to Darre Anjir WR, Ariz HPA, and Bafq PA, causing recent hybridization events. Some recent observations (A. Khajavi, personal communication) of *G. subgutturosa* in the adjacent area (Kalout‐e Abi) are congruent with this hypothesis.

### 4.4 Recent hybridization due to the anthropogenic actions

There has been at least one instance of recent hybridization of the two species due to anthropogenic action. In Shir Ahmad PA (Razavi Khorasan Province, Sabzewar), three females and one male *G. bennettii*, which were previously kept in a 25 ha fenced area in Shir Ahmad breeding center, were released to the protected area in 2006. This area is a prime habitat for *G. subgutturosa* in northeastern Iran with around 650 individuals of that species living there. Three of the four *G. bennettii* individuals died in the same year and only one female survived that was frequently observed by the game wardens (A. Khani, personal communication). In the following year that female was seen with a young. As no natural populations of *G. bennettii* live anywhere close to Shir Ahmad PA, the juvenile very likely is the offspring of the *G. bennettii* female and a *G. subgutturosa* male. Other *G. bennettii* individuals from the Shir Ahmad breeding center and their offspring were translocated to Salami Breeding Center (South Khorasan Province, Khaf), from where they were introduced to South Khorasan (Ferdous) in 2016. It is possible, that these gazelles were also in contact with *G. subgutturosa*, as some individuals of the latter could have been placed within the fenced area in Shir Ahmad (for short periods of time) for recovery from illness or after having been confiscated. Therefore, introductions and translocations, which can substantially increase the rate of hybridization in mammals in general (Allendorf et al., 2001; Rhymer & Simberloff, 1996; Vonlanthen et al., 2012) might also have led to hybridization between *G. bennettii* and *G. subgutturosa*.

## 5 CONCLUSION

Finding gazelles with mixed ancestry of *G. subgutturosa* (mitochondrial DNA) and *G. bennettii* (nuclear DNA) was very unexpected, as hybridization between these two sister species was never hypothesized before. The hybrid populations are located in central Iran, in a contact zone between the two species. *G. subgutturosa* is predominantly living in larger herds and migrating between habitats, while *G. bennettii* is more sedentary with animals living in small groups (Lerp, Wronski, Butynski, & Plath, 2013). The hybrids are phenotypically and ecologically identical with *G. bennettii* and most likely belong to the same population as pure *G. bennettii* in these areas. Especially, Khabr NP with more than 1,000 individuals is a prime habitat of *G. bennettii* in Iran and acts as a source for smaller populations of *G. bennettii* in the surrounding habitats. Although we propose that the hybridization goes back to two separate prehistoric events, as no shared haplotypes exist between hybrid and neighboring pure populations of *G. subgutturosa*, it is not entirely impossible that hybridization between *G. bennettii* and *G. subgutturosa* still occurs in areas with very low population numbers. The two species now have very different chromosome numbers, so it is possible that hybrids from recent hybridization events have reduced fertility. Therefore, keeping *G. subgutturosa* and *G. bennettii* individuals in the same fenced area in a breeding center and also introducing *G. bennettii* to the habitat of *G. subgutturosa* such as Shir Ahmad PA should be avoided under all circumstances.

For future captive breeding programs, the knowledge of these newly identified wild hybrid populations is very important. As long as the genetic makeup of these animals, especially the chromosome numbers, is not known, we strongly advise against using them in *G. bennettii* conservation programs.

## CONFLICT OF INTEREST

The authors declare that they have no competing interests.

## AUTHOR CONTRIBUTIONS

**Davoud Fadakar:** Conceptualization (equal); investigation (equal); methodology (equal); software (equal); writing – original draft (equal); writing – review and editing (equal). **Mansoureh Malekian:** Conceptualization (equal); supervision (equal); writing – original draft (equal); writing – review and editing (equal). **Mahmoud R. Hemami:** Conceptualization (equal); resources (equal); supervision (equal). **Hannes Lerp:** Conceptualization (equal); methodology (equal); resources (equal); software (equal); validation (equal). **Hamid R. Rezaei:** Conceptualization (equal); data curation (equal); resources (equal). **Eva V. Bärmann:** Conceptualization (equal); data curation (equal); funding acquisition (equal); resources (equal); supervision (equal).

## Supporting information

Table S1

## Data Availability

DNA sequences have been deposited in GenBank under the accession no: MT811607–MT811638 and MT822208–MT822269.
